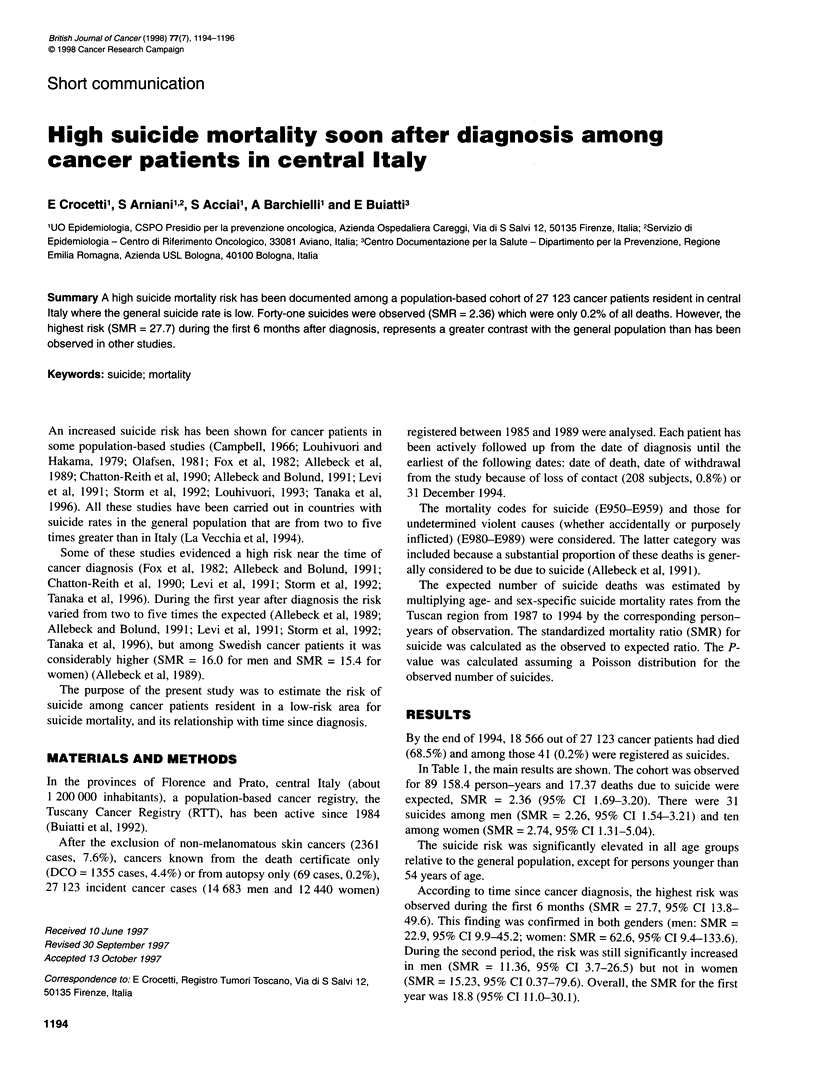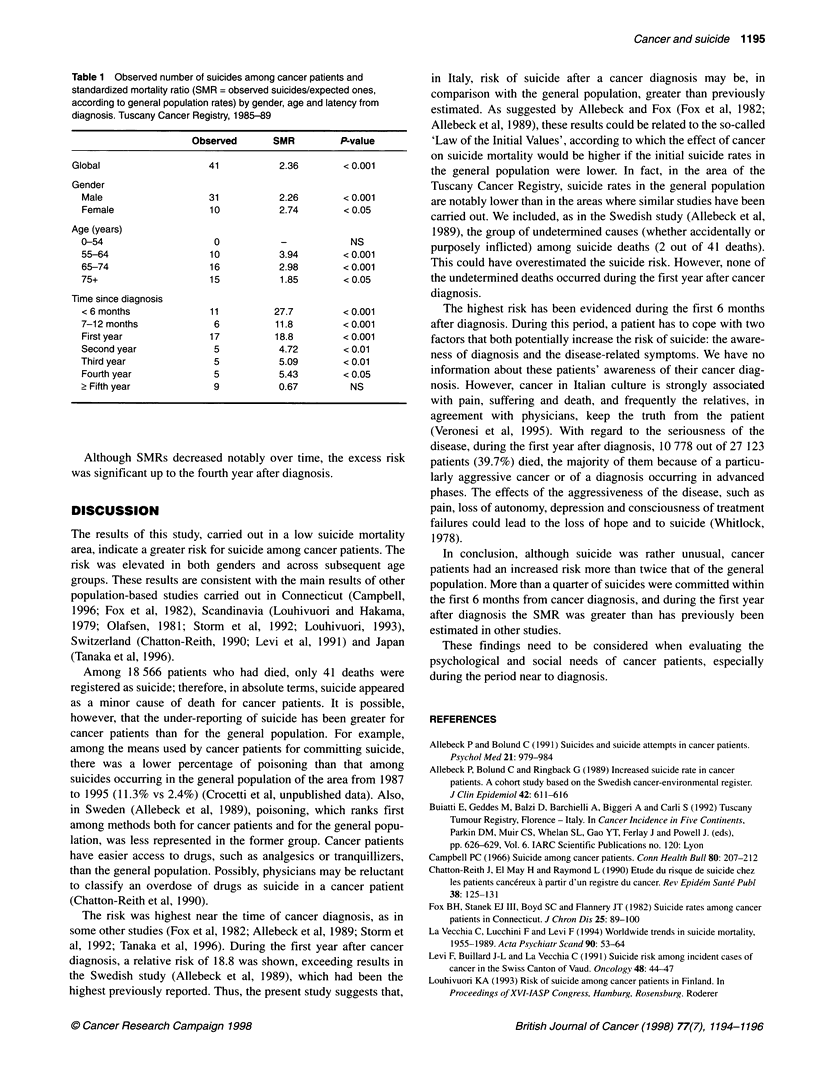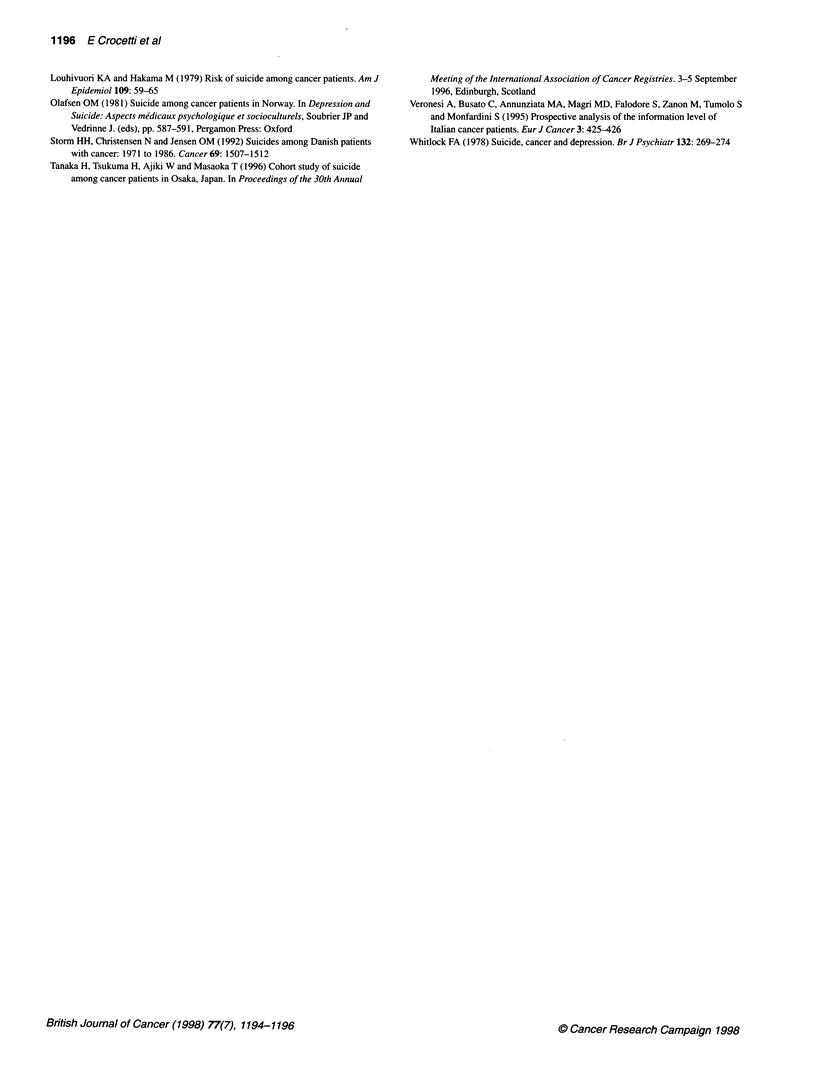# High suicide mortality soon after diagnosis among cancer patients in central Italy.

**DOI:** 10.1038/bjc.1998.199

**Published:** 1998-04

**Authors:** E. Crocetti, S. Arniani, S. Acciai, A. Barchielli, E. Buiatti

**Affiliations:** UO Epidemiologia, CSPO Presidio per la prevenzione oncologica, Azienda Ospedaliera Careggi, Firenze, Italia.

## Abstract

A high suicide mortality risk has been documented among a population-based cohort of 27 123 cancer patients resident in central Italy where the general suicide rate is low. Forty-one suicides were observed (SMR = 2.36) which were only 0.2% of all deaths. However, the highest risk (SMR = 27.7) during the first 6 months after diagnosis, represents a greater contrast with the general population than has been observed in other studies.


British Journal of Cancer (1998) 77(7), 1194–1196
© 1998 Cancer Research Campaign

Short communication

### High suicide mortality soon after diagnosis among cancer patients in central Italy

**E Crocetti[1], S Arniani[1,2], S Acciai[1], A Barchielli[1] and E Buiatti[3]**

[1]UO Epidemiologia, CSPO Presidio per la prevenzione oncologica, Azienda Ospedaliera Careggi, Via di S Salvi 12, 50135 Firenze, Italia; [2]Servizio di Epidemiologia – Centro di Riferimento Oncologico, 33081 Aviano, Italia; [3]Centro Documentazione per la Salute – Dipartimento per la Prevenzione, Regione Emilia Romagna, Azienda USL Bologna, 40100 Bologna, Italia

**Summary** A high suicide mortality risk has been documented among a population-based cohort of 27 123 cancer patients resident in central Italy where the general suicide rate is low. Forty-one suicides were observed (SMR = 2.36) which were only 0.2% of all deaths. However, the highest risk (SMR = 27.7) during the first 6 months after diagnosis, represents a greater contrast with the general population than has been observed in other studies.

**Keywords:** suicide; mortality

An increased suicide risk has been shown for cancer patients in some population-based studies (Campbell, 1966; Louhivuori and Hakama, 1979; Olafsen, 1981; Fox et al, 1982; Allebeck et al, 1989; Chatton-Reith et al, 1990; Allebeck and Bolund, 1991; Levi et al, 1991; Storm et al, 1992; Louhivuori, 1993; Tanaka et al, 1996). All these studies have been carried out in countries with suicide rates in the general population that are from two to five times greater than in Italy (La Vecchia et al, 1994).

Some of these studies evidenced a high risk near the time of cancer diagnosis (Fox et al, 1982; Allebeck and Bolund, 1991; Chatton-Reith et al, 1990; Levi et al, 1991; Storm et al, 1992; Tanaka et al, 1996). During the first year after diagnosis the risk varied from two to five times the expected (Allebeck et al, 1989; Allebeck and Bolund, 1991; Levi et al, 1991; Storm et al, 1992; Tanaka et al, 1996), but among Swedish cancer patients it was considerably higher (SMR = 16.0 for men and SMR = 15.4 for women) (Allebeck et al, 1989).

The purpose of the present study was to estimate the risk of suicide among cancer patients resident in a low-risk area for suicide mortality, and its relationship with time since diagnosis.

### MATERIALS AND METHODS

In the provinces of Florence and Prato, central Italy (about 1 200 000 inhabitants), a population-based cancer registry, the Tuscany Cancer Registry (RTT), has been active since 1984 (Buiatti et al, 1992).

After the exclusion of non-melanomatous skin cancers (2361 cases, 7.6%), cancers known from the death certificate only (DCO = 1355 cases, 4.4%) or from autopsy only (69 cases, 0.2%), 27 123 incident cancer cases (14 683 men and 12 440 women) registered between 1985 and 1989 were analysed. Each patient has been actively followed up from the date of diagnosis until the earliest of the following dates: date of death, date of withdrawal from the study because of loss of contact (208 subjects, 0.8%) or 31 December 1994.

The mortality codes for suicide (E950–E959) and those for undetermined violent causes (whether accidentally or purposely inflicted) (E980–E989) were considered. The latter category was included because a substantial proportion of these deaths is generally considered to be due to suicide (Allebeck et al, 1991).

The expected number of suicide deaths was estimated by multiplying age- and sex-specific suicide mortality rates from the Tuscan region from 1987 to 1994 by the corresponding person–years of observation. The standardized mortality ratio (SMR) for suicide was calculated as the observed to expected ratio. The *P*-value was calculated assuming a Poisson distribution for the observed number of suicides.

### RESULTS

By the end of 1994, 18 566 out of 27 123 cancer patients had died (68.5%) and among those 41 (0.2%) were registered as suicides.

In Table 1, the main results are shown. The cohort was observed for 89 158.4 person–years and 17.37 deaths due to suicide were expected, SMR = 2.36 (95% CI 1.69–3.20). There were 31 suicides among men (SMR = 2.26, 95% CI 1.54–3.21) and ten among women (SMR = 2.74, 95% CI 1.31–5.04).

The suicide risk was significantly elevated in all age groups relative to the general population, except for persons younger than 54 years of age.

According to time since cancer diagnosis, the highest risk was observed during the first 6 months (SMR = 27.7, 95% CI 13.8–49.6). This finding was confirmed in both genders (men: SMR = 22.9, 95% CI 9.9–45.2; women: SMR = 62.6, 95% CI 9.4–133.6). During the second period, the risk was still significantly increased in men (SMR = 11.36, 95% CI 3.7–26.5) but not in women (SMR = 15.23, 95% CI 0.37–79.6). Overall, the SMR for the first year was 18.8 (95% CI 11.0–30.1).

*Received 10 June 1997*
*Revised 30 September 1997*
*Accepted 13 October 1997*

*Correspondence to:* E Crocetti, Registro Tumori Toscano, Via di S Salvi 12, 50135 Firenze, Italia

**Table 1** Observed number of suicides among cancer patients and standardized mortality ratio (SMR = observed suicides/expected ones, according to general population rates) by gender, age and latency from diagnosis. Tuscany Cancer Registry, 1985–89

| | Observed | SMR | *P*-value |
|---|---|---|---|
| Global | 41 | 2.36 | < 0.001 |
| Gender | | | |
| Male | 31 | 2.26 | < 0.001 |
| Female | 10 | 2.74 | < 0.05 |
| Age (years) | | | |
| 0–54 | 0 | – | NS |
| 55–64 | 10 | 3.94 | < 0.001 |
| 65–74 | 16 | 2.98 | < 0.001 |
| 75+ | 15 | 1.85 | < 0.05 |
| Time since diagnosis | | | |
| < 6 months | 11 | 27.7 | < 0.001 |
| 7–12 months | 6 | 11.8 | < 0.001 |
| First year | 17 | 18.8 | < 0.001 |
| Second year | 5 | 4.72 | < 0.01 |
| Third year | 5 | 5.09 | < 0.01 |
| Fourth year | 5 | 5.43 | < 0.05 |
| ≥ Fifth year | 9 | 0.67 | NS |

Although SMRs decreased notably over time, the excess risk was significant up to the fourth year after diagnosis.

### DISCUSSION

The results of this study, carried out in a low suicide mortality area, indicate a greater risk for suicide among cancer patients. The risk was elevated in both genders and across subsequent age groups. These results are consistent with the main results of other population-based studies carried out in Connecticut (Campbell, 1996; Fox et al, 1982), Scandinavia (Louhivuori and Hakama, 1979; Olafsen, 1981; Storm et al, 1992; Louhivuori, 1993), Switzerland (Chatton-Reith, 1990; Levi et al, 1991) and Japan (Tanaka et al, 1996).

Among 18 566 patients who had died, only 41 deaths were registered as suicide; therefore, in absolute terms, suicide appeared as a minor cause of death for cancer patients. It is possible, however, that the under-reporting of suicide has been greater for cancer patients than for the general population. For example, among the means used by cancer patients for committing suicide, there was a lower percentage of poisoning than that among suicides occurring in the general population of the area from 1987 to 1995 (11.3% vs 2.4%) (Crocetti et al, unpublished data). Also, in Sweden (Allebeck et al, 1989), poisoning, which ranks first among methods both for cancer patients and for the general population, was less represented in the former group. Cancer patients have easier access to drugs, such as analgesics or tranquillizers, than the general population. Possibly, physicians may be reluctant to classify an overdose of drugs as suicide in a cancer patient (Chatton-Reith et al, 1990).

The risk was highest near the time of cancer diagnosis, as in some other studies (Fox et al, 1982; Allebeck et al, 1989; Storm et al, 1992; Tanaka et al, 1996). During the first year after cancer diagnosis, a relative risk of 18.8 was shown, exceeding results in the Swedish study (Allebeck et al, 1989), which had been the highest previously reported. Thus, the present study suggests that, in Italy, risk of suicide after a cancer diagnosis may be, in comparison with the general population, greater than previously estimated. As suggested by Allebeck and Fox (Fox et al, 1982; Allebeck et al, 1989), these results could be related to the so-called 'Law of the Initial Values', according to which the effect of cancer on suicide mortality would be higher if the initial suicide rates in the general population were lower. In fact, in the area of the Tuscany Cancer Registry, suicide rates in the general population are notably lower than in the areas where similar studies have been carried out. We included, as in the Swedish study (Allebeck et al, 1989), the group of undetermined causes (whether accidentally or purposely inflicted) among suicide deaths (2 out of 41 deaths). This could have overestimated the suicide risk. However, none of the undetermined deaths occurred during the first year after cancer diagnosis.

The highest risk has been evidenced during the first 6 months after diagnosis. During this period, a patient has to cope with two factors that both potentially increase the risk of suicide: the awareness of diagnosis and the disease-related symptoms. We have no information about these patients' awareness of their cancer diagnosis. However, cancer in Italian culture is strongly associated with pain, suffering and death, and frequently the relatives, in agreement with physicians, keep the truth from the patient (Veronesi et al, 1995). With regard to the seriousness of the disease, during the first year after diagnosis, 10 778 out of 27 123 patients (39.7%) died, the majority of them because of a particularly aggressive cancer or of a diagnosis occurring in advanced phases. The effects of the aggressiveness of the disease, such as pain, loss of autonomy, depression and consciousness of treatment failures could lead to the loss of hope and to suicide (Whitlock, 1978).

In conclusion, although suicide was rather unusual, cancer patients had an increased risk more than twice that of the general population. More than a quarter of suicides were committed within the first 6 months from cancer diagnosis, and during the first year after diagnosis the SMR was greater than has previously been estimated in other studies.

These findings need to be considered when evaluating the psychological and social needs of cancer patients, especially during the period near to diagnosis.

### REFERENCES

Allebeck P and Bolund C (1991) Suicides and suicide attempts in cancer patients. *Psychol Med* **21**: 979–984

Allebeck P, Bolund C and Ringback G (1989) Increased suicide rate in cancer patients. A cohort study based on the Swedish cancer-environmental register. *J Clin Epidemiol* **42**: 611–616

Buiatti E, Geddes M, Balzi D, Barchielli A, Biggeri A and Carli S (1992) Tuscany Tumour Registry, Florence – Italy. In *Cancer Incidence in Five Continents*, Parkin DM, Muir CS, Whelan SL, Gao YT, Ferlay J and Powell J. (eds), pp. 626–629, Vol. 6. IARC Scientific Publications no. 120: Lyon

Campbell PC (1966) Suicide among cancer patients. *Conn Health Bull* **80**: 207–212

Chatton-Reith J, El May H and Raymond L (1990) Etude du risque de suicide chez les patients cancéreux à partir d'un registre du cancer. *Rev Epidém Santé Publ* **38**: 125–131

Fox BH, Stanek EJ III, Boyd SC and Flannery JT (1982) Suicide rates among cancer patients in Connecticut. *J Chron Dis* **25**: 89–100

La Vecchia C, Lucchini F and Levi F (1994) Worldwide trends in suicide mortality, 1955–1989. *Acta Psychiatr Scand* **90**: 53–64

Levi F, Buillard J-L and La Vecchia C (1991) Suicide risk among incident cases of cancer in the Swiss Canton of Vaud. *Oncology* **48**: 44–47

Louhivuori KA (1993) Risk of suicide among cancer patients in Finland. In *Proceedings of XVI-IASP Congress, Hamburg, Rosensburg*. Roderer

© Cancer Research Campaign 1998

Louhivuori KA and Hakama M (1979) Risk of suicide among cancer patients. *Am J Epidemiol* **109**: 59–65

Olafsen OM (1981) Suicide among cancer patients in Norway. In *Depression and Suicide: Aspects médicaux psychologique et socioculturels*, Soubrier JP and Vedrinne J. (eds), pp. 587–591, Pergamon Press: Oxford

Storm HH, Christensen N and Jensen OM (1992) Suicides among Danish patients with cancer: 1971 to 1986. *Cancer* **69**: 1507–1512

Tanaka H, Tsukuma H, Ajiki W and Masaoka T (1996) Cohort study of suicide among cancer patients in Osaka, Japan. In *Proceedings of the 30th Annual Meeting of the International Association of Cancer Registries*. 3–5 September 1996, Edinburgh, Scotland

Veronesi A, Busato C, Annunziata MA, Magri MD, Falodore S, Zanon M, Tumolo S and Monfardini S (1995) Prospective analysis of the information level of Italian cancer patients. *Eur J Cancer* **3**: 425–426

Whitlock FA (1978) Suicide, cancer and depression. *Br J Psychiatr* **132**: 269–274

*© Cancer Research Campaign 1998*